# Influence of the Resin System and Sand Type on the Infiltration of 3D-Printed Sand Tools

**DOI:** 10.3390/ma16165549

**Published:** 2023-08-09

**Authors:** Patricia Erhard, Iman Taha, Daniel Günther

**Affiliations:** 1 Fraunhofer Institute for Casting, Composite and Processing Technology IGCV, Lichtenbergstr. 15, 85748 Garching, Germany or iman.taha@hs-aalen.de (I.T.); daniel.guenther@igcv.fraunhofer.de (D.G.); 2 Polymer Technology, Aalen University of Applied Science, Beethovenstr. 1, 73430 Aalen, Germany

**Keywords:** binder jetting, sand mold, resin infiltration, vacuum thermoforming, fiber-reinforced composites, tool

## Abstract

Binder jetting is a highly productive additive manufacturing (AM) method for porous parts. Due to its cost-effectiveness, it is used for large components and quantities ranging from prototyping to series production. Post-processing steps like sintering or infiltration are common in several applications to achieve high density and strength. This work investigates how 3D-printed sand molds can be infiltrated with epoxy resins without vacuum assistance to produce high-strength molds for thermoforming applications. Specimens 3D-printed from different sand types are infiltrated with resins of different viscosity and analyzed for infiltration velocity and depth. The infiltration velocities corresponded well with the correlation described in Washburn’s equation: The resins’ viscosities and the saturation level were decisive. Amongst the investigated sand types commonly used in foundries, sand type GS19 was found most suitable for infiltration. However, the sand type proved to be a less relevant influencing factor than the resins’ viscosities and quantities applied. Infiltration of topology-optimized 3D-printed sand tools up to a wall thickness of 20 mm for thermoforming applications was found to be feasible.

## 1. Introduction

Creating reliable tooling poses significant challenges as they frequently possess intricate designs, necessitating precise dimensional accuracy, strength, excellent surface finish, and wear resistance [1]. Often lightweight tools are necessary to facilitate mounting and dismantling jobs. The traditional method of manufacturing tooling, especially for prototyping or small series, can be both time-consuming and costly [2]. Machined tools are often not economically viable due to the high investment costs and lead times, especially in low-volume production. Rapid tooling is defined as tooling that can be provided with reduced lead time compared to traditional manufacturing [3]. In addition to the advantage of rapid production and a potentially fully digitized production line [4], technological reasons may justify using AM methods for tooling applications. For instance, AM enables integrating conformal cooling or heating channels near the mold surface for more uniform and efficient heat dissipation [5].

According to the Wohlers Report 2022, 6.8% of organizations using AM parts are engaged in tooling applications related to polymer and sand patterns, cores, and molds, and 3.5% in metal molds or dies [3].

Typical applications for metal tools are injection molding and die casting. Brotan et al. [6] suggest a Laser Powder Bed Fusion (L-PBF) process to create metal tools featuring complex gradient structures, enhanced thermal fatigue resistance, and precise thermal management from steel powder. Asnafi [7] values the potential of L-PBF toolings as the greatest for hot working and injection molding due to the achievable operation performance. Efficient design, e.g., optimized lattice structures [8] and a microstructure adjustable on the process side [9], are technological benefits. However, due to their high costs and the competition with conventional manufacturing, additively manufactured metal tools are mostly not economically viable. Although additive manufacturing can reduce manufacturing costs by up to 14% compared to conventional milling methods, the high costs of metal powder (167%) offset the savings [10]. For example, Leal et al. [11] calculated significantly higher total production costs for stamping tool inserts fabricated by direct metal AM compared to such produced from laminated steel or by lost foam casting.

Various studies have focused on utilizing additive manufacturing to fabricate tools from polymer materials. In the medical field, successful demonstrations and implementations were carried out for thermoforming plastic structures using molds made from calcium sulfate and gypsum powders and binder jetting technology [12,13]. Junk et al. [10] explored the application of inkjet technology for thermoforming mold fabrication using polymer gypsum. A case study involving the production of an ABS fairing for an uncrewed aerial vehicle was conducted, aiming to integrate vacuum channels during manufacturing without the need for additional post-processing like drilling. The study addressed technical challenges such as demolding undercuts and tool separation. The results indicated that the tool exhibited sufficient strength for the subsequent thermoforming process. Another advantage of additive manufacturing highlighted in this study was the flexibility in designing both the overall geometry and internal structure to influence strength and other physical properties for optimized vacuum guidance [12,14]. Due to its projected annual growth rate, composite tooling, e.g., dies and molds for lay-ups, vacuum, stretch, and thermal forming, is of growing interest [15]. Warden [16] investigated the utilization of Fused Deposition Modeling (FDM) for manufacturing tools used in compression molding of thermoplastic multiaxial prepreg systems. The author proposed a low-temperature curing cycle to minimize the thermal degradation of the FDM tooling. Bere et al. [17] processed Carbon-Fiber-Reinforced Polymers (CFRP) using vacuum bagging and oven curing, employing an FDM mold made of polylactic acid (PLA) or acrylonitrile butadiene styrene (ABS). Hassen et al. [18] suggested using the extrusion-based Big Area Additive Manufacturing (BAAM) process to produce CFRP tools for subsequent molding purposes. The key advantages included higher throughput (~16,400 cm^3^/h) and the ability to process material in granule form [18,19].

Three-dimensional-printed sand cores have already been implemented into the series production of metal castings [20]. The binder jetting process and the properties of sand cores relevant to the casting process are studied frequently. Sand cores must be strong enough to withstand the thermal and mechanical loads during casting but still be removable from the casting after the solidification of the metal [21]. Typical flexural strengths are between 2.5 and 5 MPa [22]—strengths allow only single-use as lost tooling in gravity or low-pressure die casting. Infiltration of binder jetted ceramic parts is a common process for strengthening and densification [23]. The strength of ceramic preforms formed by AM can be adjusted by impregnation with a ceramic suspension and subsequent sintering [24,25]. Epoxy infiltration is another option for post-processing to strengthen printed parts. Garzón et al. [26] achieved an increase in specimens’ flexural strength of ~447% with epoxy infiltration. Maravola et al. [27] examined the mechanical and thermal properties of sand specimens infiltrated with epoxy by vacuum infiltration and demonstrated the feasibility of infiltrated sand molds for composite toolings. A CFRP part was manufactured by hand lay-up on the tooling.

**Figure 1:**
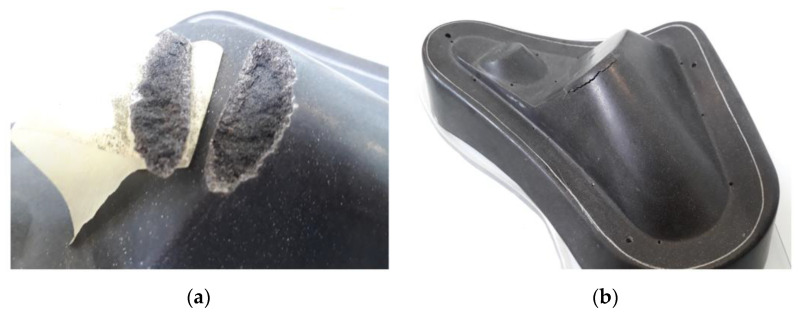
Damage analysis on a 3D-printed and resin-infiltrated thermoforming tool. (**a**) Detail of the spalling, (**b**) overview showing the location of damage.

In [28], the authors introduced the basic idea of tools fabricated via binder jetting for vacuum thermoforming fiber-reinforced polymer composites. The authors claim that silica sand proves to be an efficient and cost-effective material for this application, especially due to its low thermal conductivity. Due to the process loads in vacuum forming, this application also requires a post-treatment to improve the mechanical performance of sand tools. Even though it is known that vacuum assistance improves the infiltration of specimens [29,30], the goal is to develop sand tools that are free from vacuum buildup, which would allow for easier and more versatile processing and a wider range of possible sizes. Vacuum resin infusion is a standard process in composite technology. However, no prior work is known to the authors focusing on the infiltration range and dynamics during epoxy infiltration of 3D-printed toolings without vacuum assistance. The first application tests of a spray-infiltrated tool in thermoforming showed spalling within ten production cycles (Figure 1). The epoxy infiltrate only penetrated a few millimeters into the material’s surface, causing too-high shear stresses on the interface between the surface strengthened by infiltration and the untreated core material during thermoforming.

**Figure 2:**
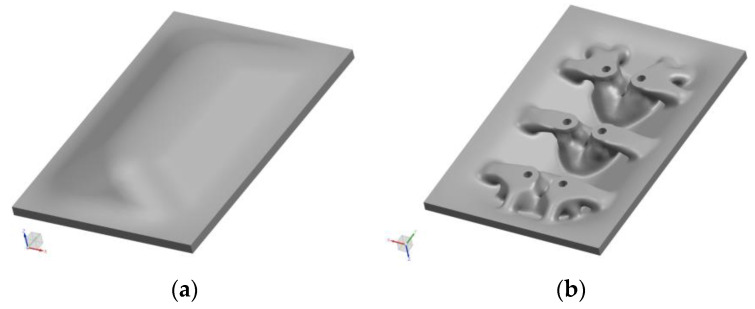
Design of a topology-optimized mold with a maximum wall thickness of 20 mm. (**a**) Top view, (**b**) bottom view.

This observation gives the impulse to investigate the infiltration process of 3D-printed sand substrates with epoxy resins. It is intended to determine which resin and sand material systems are the most suitable for infiltration and if full infiltration is feasible to realize no-longer-massive (Figure 1) but rather high-strength and resource-efficient topology-optimized molds. Figure 2 shows an example of a tool concept: The top layer, the molding geometry, is designed as a shell with a designated wall thickness (offset to the molding surface, Figure 2a). The shell is supported by a load-bearing structure that transfers loads to the machine base (Figure 2b). The process knowledge of infiltration generated herein and material data on infiltrated sand determined in parallel (sand specimens fully infiltrated with epoxy resin showed compressive strengths >100 MPa) will empower the correct setting of boundary conditions for realizing topology-optimized tools by binder jetting and subsequent epoxy infiltration for thermoforming CFRP parts.

## 2. Theory on Infiltration of Porous Substrates

The infiltration of a substrate by a liquid or the penetration of a liquid in a porous substrate is preceded by a spreading and wetting phase [31]. For predicting the infiltration behavior, the porosity of the substrate bulk material plays a crucial role in infiltration. The more porous a material is and the less tortuous a porous structure is shaped, the lower its flow resistance and the greater its permeability [32]. Infiltration can be promoted by controlling the inherent material porosity [33]. Commonly, porosity is assumed to be a network of capillaries. For simplification, equations describing the capillary flow in parallel cylinder tubes are used [34]. Capillary pressure is the driving force for the migration of liquids in porous substrates. The flow of receding liquids under capillary pressure can be described by Washburn’s equation (Equation (1)).

(1) $$h^{2}=\frac{\gamma \cdot cos\theta }{2\eta }r\cdot t$$

with the height of a liquid column h, the surface tension γ, the contact angle θ, the pore radius r, time t, and the viscosity η. By rearranging the equation, the infiltration rate during capillary flow can be predicted as by Equation (2).

(2) $$\frac{dh}{dt}=\frac{\gamma \cdot r\cdot cos\theta }{4h\cdot \eta }$$

A liquid’s retraction rate is thus proportional to the radius of a capillary, the liquid’s surface tension, and the cosine of the contact angle, and inversely proportional to the liquid’s viscosity and the height of a liquid column that is already filled with liquid. Thus, the fluid’s and substrate’s properties, the wettability of the substrate material by the liquid, and the filling height of the pore columns determine the infiltration behavior [35].

Denesuk et al. [36] developed a model for the drainage of liquid droplets during the infiltration of porous materials and distinguished between a model with constant and decreasing drawing area. The depletion process was found faster by a factor of 9 for a decreasing drawing area. When depositing small droplets (~60 µm in diameter as typical for binder jetting applications) on porous powder beds, spreading and infiltration are superimposing and cease within the range of milliseconds. Particle size (or pore size) was found to affect the infiltration time and spreading extension [37]. Holman [38] observed a filtering mechanism leading to a loss of binder molecule concentration with increasing infiltration depths.

When studying infiltration, it is important to consider the initial conditions, particularly the local fluid-powder ratios. In 3D printing, very small droplets are deposited on powder bulks. However, the infiltration kinetics fundamentally differ when comparing conventional deposition of powders to high-density powder beds that may be, e.g., realized by slurry-based 3D printing [39]. This study focuses on the absorption of larger volumes of liquid, resulting in mostly highly saturated areas. For comparison, droplets of microliter size are investigated.

## 3. Materials and Methods

### 3.1. Materials

Various sand types are considered in this study to investigate the effect of the respective chemical composition, particle size, and morphology on the penetrability of binder jetted specimens. All systems are commercially available as raw material for 3D printers or print-on-demand products in already shaped condition. Three different particle size distributions of natural silica sand are investigated, as further detailed in Table 1.

Specimens are fabricated from these sands using binder jetting with organic binders (phenolic resin for chromite sand, furanic resin for the others) at standard parameters for 3D printing of sand molds and cores. Three different infiltration media are selected based on their strongly differing viscosities (Table 2). For ease of reading, the products are referred to as CH83-10, IH16, and LH28.

**Figure 3:**
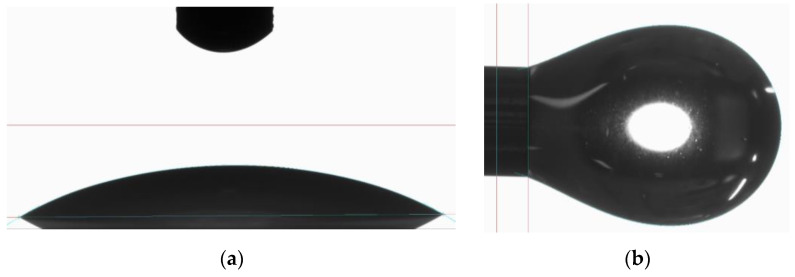
Optical determination of (**a**) contact angle and (**b**) surface tension using OCA 25 measurement device.

Surface tension is determined for droplets of 6 µL (CR83-10), 5 µL (IH16), or 6 µL (LH28) using the optical contact angle goniometer and drop shape analysis system OCA 25 (DataPhysics Instruments GmbH, Filderstadt, Germany) (Figure 3). A droplet volume of 8 µL is used for the contact angle measurement.

Table 3 shows the results of the contact angle and surface tension measurements. It can be seen that the values vary only slightly between the resin systems.

### 3.2. Specimen and Experimental Method

**Figure 4:**
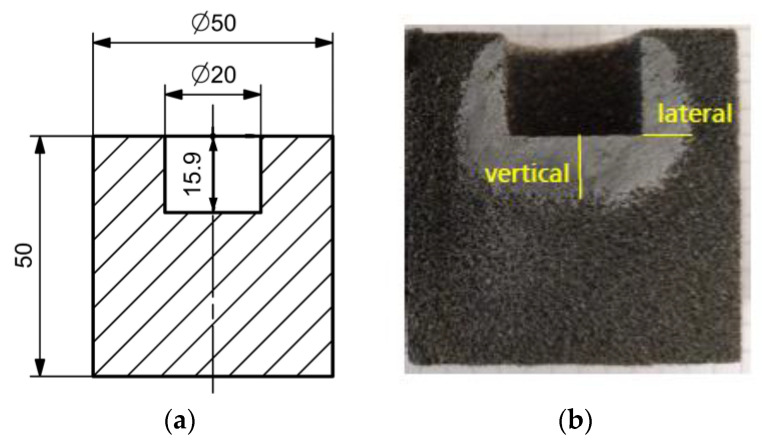
Specimen design for investigation of resin infiltration. (**a**) Dimensions, (**b**) infiltrated specimen cut in half and visualization of the determination of vertical and lateral infiltration.

Resin infiltration is investigated using the specimen design illustrated in Figure 4. It is a solid cylinder with a cylindrical pocket of 5 mL volume, which can be filled with the infiltration medium.

All specimens are 3D printed in the upright direction, as illustrated in Figure 4, using a VX1000 printer (voxeljet AG, Friedberg, Germany) at voxeljet’s 3D print-on-demand service’s industrial standard parameters at an accuracy of ±0.1% and cleaned from loose sand with the help of pressurized air. The infiltration experiments are conducted at room temperature (20 ± 1 °C). The two-component resins are mixed immediately before the infiltration tests according to the suppliers’ product instructions in quantities of 100 g.

Three infiltration steps are performed for each infiltration experiment: Using syringes, the test specimens’ pockets are filled with 5 mL of properly mixed resin. After the infiltration medium is completely retracted, another 5 mL is added. The same procedure is repeated once the second amount of liquid is retracted. The time to complete retraction, i.e., the point at which the liquid level drops below the pocket’s flat bottom, is observed visually by the laboratory technician. It is recorded using an electronic watch with a stopwatch function with an estimated reading accuracy of 5 s. The one-factor-at-a-time (OFAT) method is chosen for experimental design. All available particle systems are tested for their infiltrability with the resin CH83-10. Moreover, the industry standard for 3D sand printing, the GS14 silica sand system, is tested with all three resin systems. All experiments are conducted with a sample size of three.

### 3.3. Examination Methods

For comparison between the microscopic and macroscopic behavior, infiltration is investigated on a micro-scale using the optical contact angle goniometer and drop shape analysis system OCA 25 (DataPhysics Instruments GmbH, Filderstadt, Germany). Here, only 15 g of each resin is mixed prior to the measurement, and droplets of 6 µL (CR83-10, IH16) or 8 µL (LH28) are formed and deposited on flat 3D printed substrates using the same materials as given in Table 1. Each material combination is examined on three samples (full-factorial experiment). The infiltration time of a single droplet is determined by recording the deposition process with a built-in camera. The contact angle cannot be determined on the porous substrate since it strongly depends on the surfaces’ properties and changes rapidly after the droplet’s deposition. The infiltration velocity given is the average infiltration velocity in µL/s for the single droplet investigation.

Analogously, with respect to the macroscopic examination setup, the infiltration velocity is determined as the average rate at which resin is drawn into a sand specimen during the first, second, and third infiltration steps (5 mL, 10 mL, and 15 mL, respectively).

The specimens are cut in half using the precision cut-off machine Brillant 220 (QATM, Mammelzen, Germany) and photographed. Images are processed using ImageJ image processing software [40] to examine lateral and vertical infiltration. Figure 4b shows an exemplary result. From the contrast between the infiltrated and non-infiltrated area, the infiltration depth can be determined with an accuracy of ±1 mm. The expected dimensional errors are composed of the reading inaccuracy, the deviation during 3D printing (±0.1%), and the deviation due to an imprecise cutting process. The reading inaccuracy is considered to be the most decisive error. Its impact on the result is minimized by measuring the infiltration area on the six halves of the three samples of each experiment.

## 4. Results and Discussion

### 4.1. Infiltration Velocity

**Figure 5:**
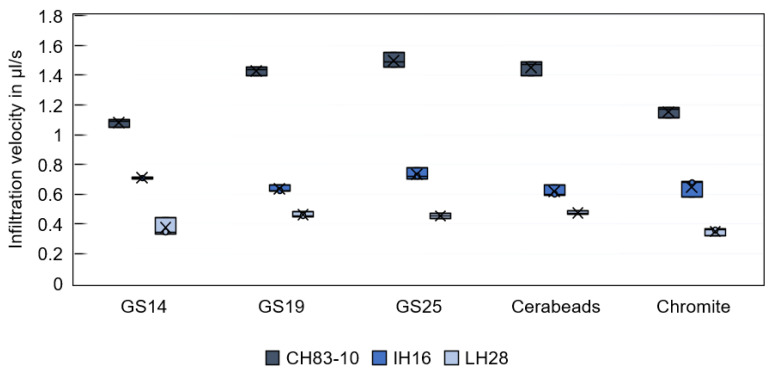
Infiltration velocity of single µL-sized resin droplets in substrates binder jetted with different sand types.

Figure 5 displays the infiltration velocity of a single resin droplet. As expected, the viscosity of the resin proved to determine how fast the resin infiltrates. A droplet of the low-viscosity resin CH83-10 is found to infiltrate into the sand substrates at an average rate of 1.3 µL/s, while the medium-viscosity resin IH16 shows an average infiltration velocity of 0.7 µL/s, and the high-viscosity resin only 0.4 µL/s. When using the resins CH83-10 or LH28, it is observed that relatively low infiltration velocities are achieved with the substrates printed from GS14 and chromite sand. Otherwise, due to the small droplet size and the optical evaluation method, the infiltration velocities cannot be reliably distinguished.

**Figure 6:**
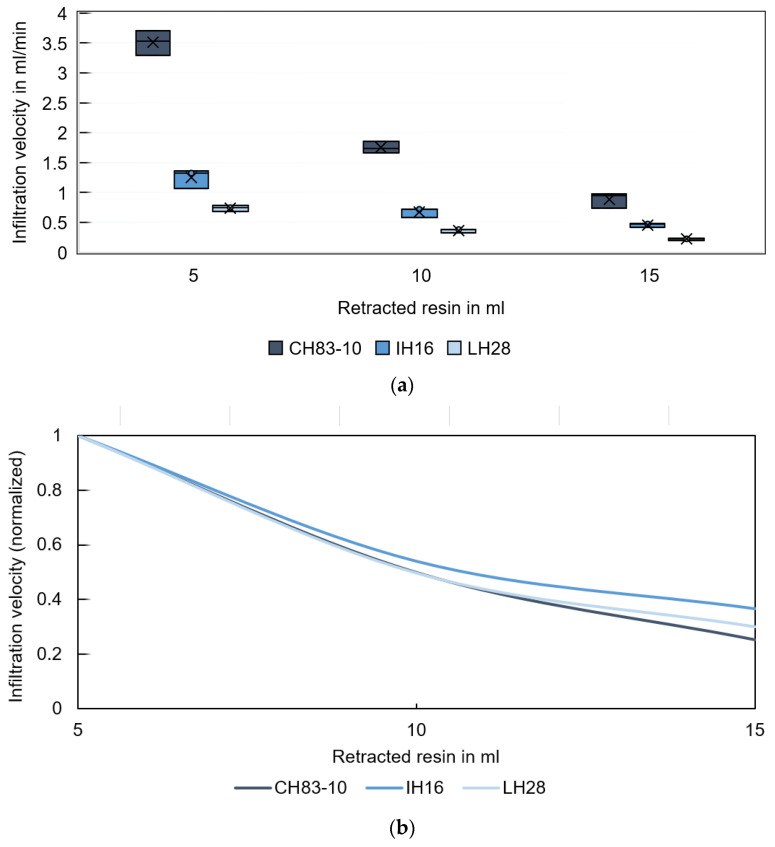

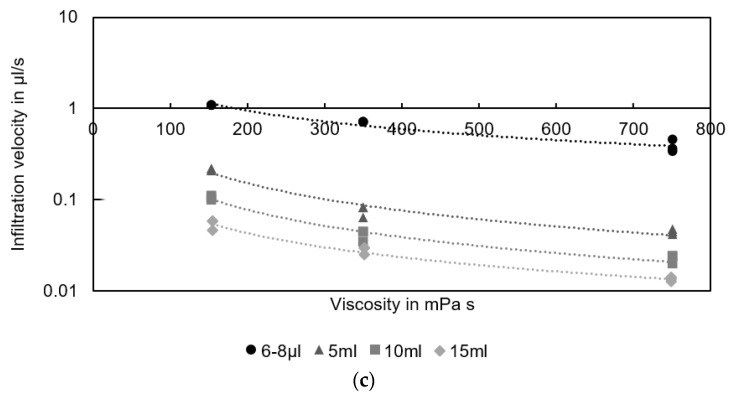
(**a**) Average infiltration velocity of different resins during the three infiltration steps of specimens printed from GS14 sand. (**b**) Infiltration progress normalized to the initial values. (**c**) Infiltration velocity as a function of resin viscosity for different infiltration volumes with power trend lines.

Figure 6a shows the infiltration velocity of different resins during the gradual infiltration of GS14 specimens using larger volumes of infiltration fluid. The first 5 mL are infiltrated into the substrate at an average rate of 3.5 mL/min for CH83-10, 1.3 mL/min for IH16, and only 0.7 mL/min for LH28. Between 5 and 10 mL, the resin CH83-10 is infiltrated at half the rate (Figure 6b). From 10 to 15 mL, the velocity decreases to about 30% of the initial infiltration velocity. With increasing saturation, the infiltration velocity declines significantly. The infiltration velocities are found to be in different orders of magnitude when comparing single droplet infiltration (in the µL range) and high saturation resin infiltration (in the ml range). Since a single droplet’s spreading and infiltration on a porous substrate superimpose [36] and spreading is typically completed after less than 100 µs [31], this observation coincides with the expectations. Investigations conducted in hydrology and soil science confirm an influence of the ponding status (the condition before and after a liquid level forms upon a substrate [41]) and differing fluid pressure head values or fluid ponding depths on the infiltration rates [42,43]. Figure 6c shows the relationship between infiltration velocity and the resin viscosities for different infiltration volumes with power trend lines. It can be seen that the infiltration velocity is a function of viscosity and saturation. This observation is consistent with the correlation described in Equation (2).

**Figure 7:**
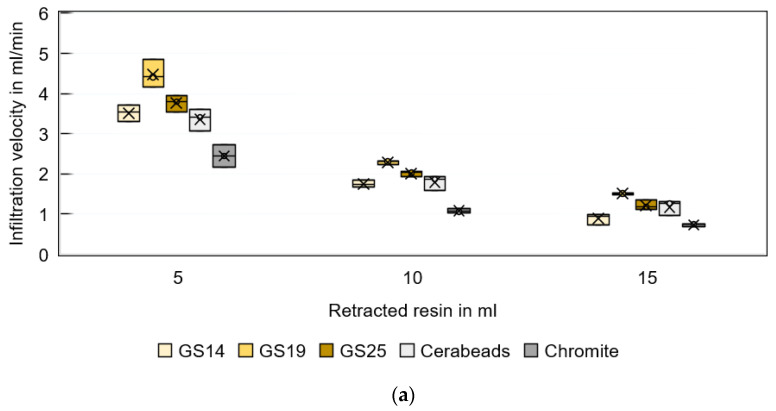

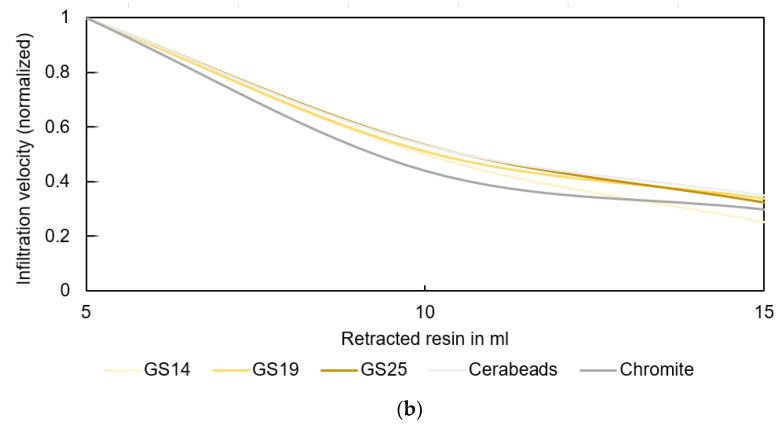
(**a**) Average infiltration velocity of resin CH83-10 during the three infiltration steps of specimens printed from different sand types. (**b**) Infiltration progress normalized to the initial values.

Figure 7a further compares the infiltration velocity using different 3D-printed sand substrates and the low-viscosity resin CH83-10. The infiltration velocity is found to vary between 2.4 and 4.5 mL/min for the first 5 mL batch, between 1.0 and 2.3 mL/min for the second 5 mL batch, and between 0.7 and 1.5 mL/min for the third 5 mL batch. A 47–56% reduction in infiltration velocity during the second batch is observed for all sand materials. The third batch correlates with an infiltration velocity of only 25–35% of the initial batch (Figure 7b).

The lowest infiltration velocity is observed for chromite sand. This can be attributed to the size and shape of the particles, which have a crucial effect on the flow of powders [44,45]. Flowability is a decisive property for 3D-printing materials to enable homogeneous and dense powder beds [46]. Deposition of poorly flowable powders often results in unevenly packed layers. Recknagel et al. [47] showed micrographs of the grain surface structures and shapes of different sand materials. A pronounced angular shape is characteristic of chromite sand, while Cerabeads are almost round, and silica sands have rounded edges. Due to their angular shape and relatively low flowability, chromite sands are known to be the most difficult to process and have the highest probability of defect formation during 3D printing.

**Figure 8:**
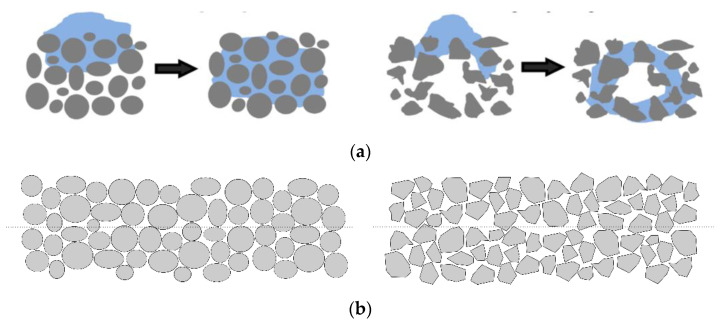
Schematics showing the effect of the uniformity of the powder distribution on the infiltration behavior. (**a**) Schematic representation of flow front propagation in a homogeneous (**left**) and heterogeneous (**right**) powder. Reproduced under terms of the CC-BY license. [31] Copyright 2021, Elsevier. (**b**) Schematic illustration of the microstructure expected at the layer interfaces in binder jetting: homogeneous structure due to flowable, round grains (**left**), non-uniform packing, and a clearly visible layer interface with macro-voids (**right**).

Perfectly matched powder deposition and printing parameters, combined with highly flowable powders, result in homogeneous random close packing, whereas irregular packing can cause macro-voids to be preferentially located at the layer interfaces (Figure 8). These can cause a rapid jump in the pore radius and thus may locally inhibit fluid drainage. The resin must flow around an obstacle to advance further in the powder bed, ultimately leading to a distorted penetration profile and increased infiltration time [48,49].

The highest infiltration velocity in this study is observed in combination with GS19 silica sand. This is noteworthy because GS19 sand has a comparable grain morphology to GS14 and GS25 (i.e., edge-rounded) and a higher grain size than GS14 but a lower grain size than GS25. The observed behavior cannot be explained by the known properties of the sand or by the equations listed above. Since the overall density of the printed parts increases with coarser grains, it is suspected that the 3D printing machine parameters (e.g., powder deposition speed) are better suited to the GS19 material system than GS14 or GS25 in terms of interlayer defects. Further investigation is needed to prove this hypothesis.

### 4.2. Infiltration Depth

Ensuring complete penetration and avoiding interfaces between infiltrated and non-infiltrated volumes is important to ensure sufficient strength and durability of sand tooling produced by binder jetting and subsequent resin infiltration. Therefore, the infiltration and propagation of the resin in the sand substrate is investigated.

**Figure 9:**
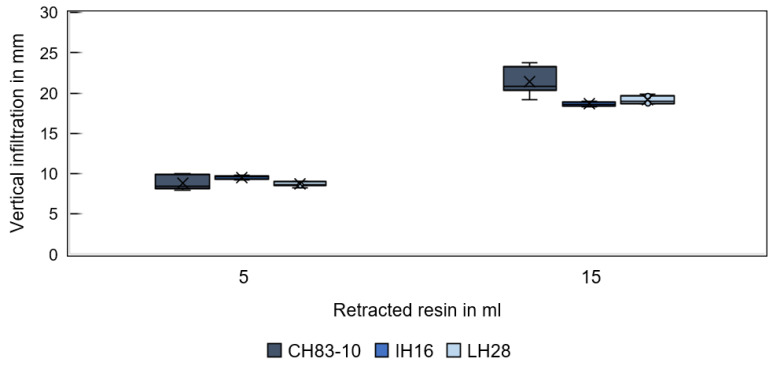
Vertical infiltration depth of different resins in specimens printed from GS14 sand for a 5 and 15 mL resin volume.

**Figure 10:**
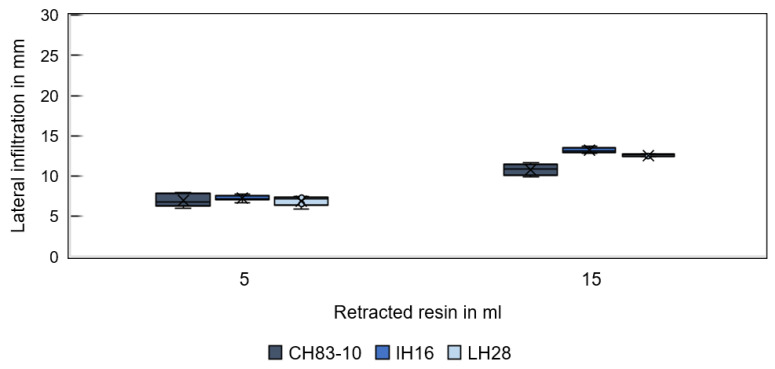
Lateral infiltration spread of different resins in specimens printed from GS14 sand for a 5 and 15 mL resin volume.

Figure 9 shows the infiltration depth in GS14 substrates for different resins and fluid volumes in the vertical direction (corresponding to the direction of gravity) and Figure 10 in the lateral direction. Regardless of the resin type, the first rapidly withdrawn resin volume of 5 mL spread equally far. Conversely, differences are observed for a total retracted resin volume of 15 mL. A more pronounced infiltration depth is observed for resin CH83-10 compared to the other two resins, which behave similarly. The increased vertical infiltration depth of CH83-10 is attributed to the significantly higher infiltration velocity compared to the other resins and, in turn, to the lower resin viscosity. Rapid vertical infiltration negatively affects lateral infiltration in the radial direction (through the pocket walls). Instead, the center from which the liquid is distributed by capillary forces may be shifted to a lower point within the specimen. The reason for inhomogeneous spreading is assumed to be the specimens’ geometry in combination with the resins’ properties. Gravity is not expected to play a role. CH83-10 infiltrates about 21 mm in the vertical direction, while IH16 and LH28 infiltrate only to a depth of about 19 mm. Therefore, resin CH83-10 may be advantageous for rapid infiltration and deep penetration.

**Figure 11:**
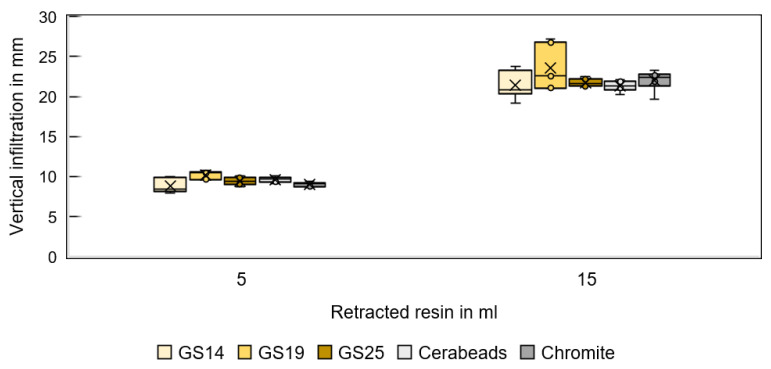
Vertical infiltration depth of resin CH83-10 in specimens printed from different sands for a 5 and 15 mL resin volume.

**Figure 12:**
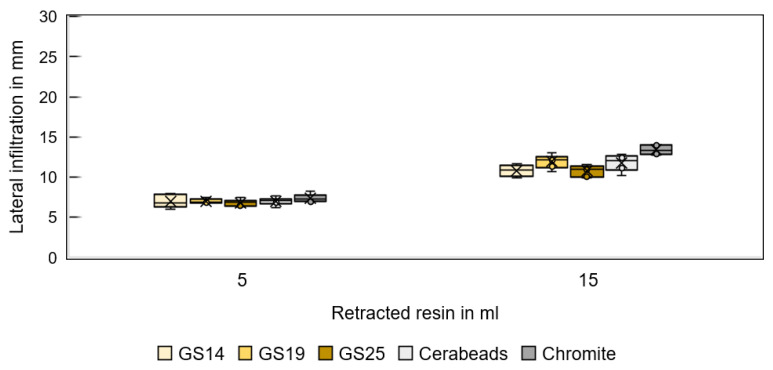
Lateral infiltration depth of resin CH83-10 in specimens printed from different sands for a 5 and 15 mL resin volume.

Figure 11 shows the infiltration depth of resin CH83-10 for different sands and fluid volumes in the vertical direction and Figure 12 in the lateral direction. GS19 has the deepest vertical penetration, and chromite the deepest lateral infiltration.

Thus, the vertical infiltration depth is found to correlate with the infiltration velocity. The investigations showed that the sand type influences the infiltration depth. However, within the sand types studied, the resin type is found to have a greater influence on the infiltration velocity and vertical depth. This is expected because the porosities of the printed substrates were relatively consistent while the resin properties vary widely.

## 5. Conclusions

Among the resin properties, viscosity was found to have the greatest effect on infiltration. In this study, infiltration resins were used that did not differ significantly in surface tension and contact angle but had widely varying values of mixed viscosity (155, 350, and 750 mPa∙s). The infiltration velocities determined were in good agreement with Washburn’s equation (Equation (2)). Resin CH83-10 has a viscosity of approximately 21% of that of LH28 and were found to infiltrate at 5 times the rate of LH28. Resin IH16 exhibits a viscosity of about 47% and infiltrated at almost twice the rate. It is difficult to determine the pore radius and capillary length in the printed sand bodies that contribute to the infiltration velocity (Equation (2)). However, porosities and grain sizes of the sand specimens used in this study did not vary greatly (Table 1). Thus, Washburn’s equation may be applied to predict the infiltration behavior of resin in 3D-printed sand specimens.

Rapid resin retraction is essential for an efficient and effective infiltration process in the production environment. It allows for deeper penetration within the pot life of a resin, which can be as low as 60 min, and makes it easier to apply resin efficiently without risking the formation of resin noses.

Silica sand was found to be a suitable material system for resin-infiltrated tools. Epoxy resin infiltration may provide sufficient strength and durability in a thermoforming process. In this study, GS19 sand performed best in terms of infiltration. However, all sand specimens were infiltrated to a depth greater than 20 mm. GS14 or even finer sands could be used for higher surface finishes in binder jetting.

Since sand specimens fully infiltrated with epoxy resin can achieve compressive strengths exceeding 100 MPa, the infiltration of topology-optimized structures using the approach proposed herein can result in highly durable thermoforming molds—a more versatile and resource-efficient manufacturing alternative compared to vacuum infusion.

## Figures and Tables

**Table 1:** Sand materials with their composition, morphology, average grain size, and porosity in the binder jetted state.

Identification	Particulate Material	Manufacturing Method	Morphology	Medium Grain Size in µm	Printed Part Porosity in %
GS14 ^1^	Quartz sand	Mined & ground	Edge-rounded	130	51.7
GS19 ^1^	Quartz sand	Mined & ground	Edge-rounded	196	50.5
GS25 ^1^	Quartz sand	Mined & ground	Edge-rounded	242	49.4
Cerabeads ES650 ^2^	Sintered mullite	Sintered	Round	200	49.8
Chromite ^3^	Chromite sand	Ground	Angular	210	49.0

^1^ Strobel Quarzsand GmbH, Freihung, Germany. ^2^ Hüttenes-Albertus Chemische Werke GmbH, Düsseldorf, Germany. ^3^ Possehl Erzkontor GmbH & Co. KG, Lübeck, Germany.

**Table 2:** Infiltration media with their characteristics according to their datasheets.

Identification Resin/Hardener	Mixed Viscosity @25 °C in mPa s	Pot Life @100 g, 25 °C in min	Compressive Strength in MPa
Biresin CR83/CH83-10 ^4^	155	300	131
IH16/Fast hardener ^5^	350	55	135
LH28-1/TM ^5^	750	300	95

^4^ Sika Deutschland GmbH, Stuttgart, Germany. ^5^ ebalta Kunststoff GmbH, Rothenburg ob der Tauber, Germany.

**Table 3:** The resins’ contact angle and surface tension measured using the OCA 25 as an average of 5 replicates. Droplet volumes for determination of surface tension 5–6 µL, for contact angle 8 µL. Stabilization time 1 min.

	CH83-10	IH16	LH28
Average contact angle in [°]	27.5	31.0	29.4
Average surface tension (average tolerance) in [mN/m]	30.6 (0.11)	29.3 (0.33)	35.8 (0.25)

## Data Availability

Not applicable.
